# Optimizing the Performance of Pure ALOHA for LoRa-Based ESL

**DOI:** 10.3390/s21155060

**Published:** 2021-07-26

**Authors:** Malak Abid Ali Khan, Hongbin Ma, Syed Muhammad Aamir, Ying Jin

**Affiliations:** School of Automation, Beijing Institute of Technology, Beijing 100081, China; 3820202079@bit.edu.cn (M.A.A.K.); 3820181051@bit.edu.cn (S.M.A.); jinyinghappy@bit.edu.cn (Y.J.)

**Keywords:** LoRa, machine learning, electronic shelf labels, aloha, spreading factor, bandwidth

## Abstract

(1) Background: The scientific development in the field of industrialization demands the automization of electronic shelf labels (ESLs). COVID-19 has limited the manpower responsible for the frequent updating of the ESL system. The current ESL uses QR (quick response) codes, NFC (near-field communication), and RFID (radio-frequency identification). These technologies have a short range or need more manpower. LoRa is one of the prominent contenders in this category as it provides long-range connectivity with less energy harvesting and location tracking. It uses many gateways (GWs) to transmit the same data packet to a node, which causes collision at the receiver side. The restriction of the duty cycle (DC) and dependency of acknowledgment makes it unsuitable for use by the common person. The maximum efficiency of pure ALOHA is 18.4%, while that of slotted ALOHA is 36.8%, which makes LoRa unsuitable for industrial use. It can be used for applications that need a low data rate, i.e., up to approximately 27 Kbps. The ALOHA mechanism can cause inefficiency by not eliminating fast saturation even with the increasing number of gateways. The increasing number of gateways can only improve the global performance for generating packets with Poisson law having a uniform distribution of payload of 1~51 bytes. The maximum expected channel capacity usage is similar to the pure ALOHA throughput. (2) Methods: In this paper, the improved ALOHA mechanism is used, which is based on the orthogonal combination of spreading factor (SF) and bandwidth (BW), to maximize the throughput of LoRa for ESL. The varying distances (D) of the end nodes (ENs) are arranged based on the K-means machine learning algorithm (MLA) using the parameter selection principle of ISM (industrial, scientific and medical) regulation with a 1% DC for transmission to minimize the saturation. (3) Results: The performance of the improved ALOHA degraded with the increasing number of SFs and as well ENs. However, after using K-mapping, the network changes and the different number of gateways had a greater impact on the probability of successful transmission. The saturation decreased from 57% to 1~2% by using MLA. The RSSI (Received Signal Strength Indicator) plays a key role in determining the exact position of the ENs, which helps to improve the possibility of successful transmission and synchronization at higher BW (250 kHz). In addition, a high BW has lower energy consumption than a low BW at the same DC with a double-bit rate and almost half the ToA (time on-air).

## 1. Introduction

**Figure 1:**
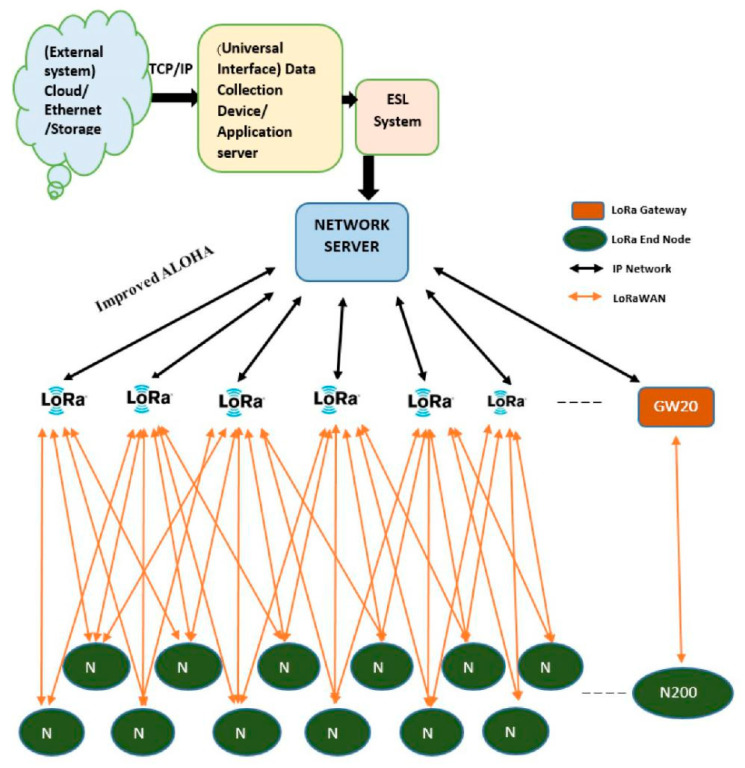
Overview of LoRa-based ESL.

Electronic shelf labels (ESL) involves wireless communication to update content on shelving displays frequently. An electronic shelf label is a device for the display of product rates, sales promotions, and different records utilized by retailers to replace traditional paper price tags. They are typically attached to the front fringe of a retail shelf and use a liquid crystal display or E-paper technology to show the statistics. They follow the dynamic pricing scheme to support fast-changing prices for synchronizing the product cost across the country, region, and city. They are suitable for retail stores including supermarkets, national utility store chains, and mega marts. They are more convenient for product tracking. Promotions and advertisements are easily handled, and the colorful display is attractive to customers. The long battery life and modification of software make them mandatory for future use. The LoRa-based ESL includes a network server, gateways (GWs), and node price that is connected to an external source by using TCP/IP. The user interface provides a platform for the ESL system to understand and manage the storage or updates of the shelves [1,2]. Network optimization plays an important and sometimes critical role in the Internet of Things, where issues such as network routing, energy conservation, congestion control, heterogeneity, scalability, reliability, quality of service, and/or security may be considered [3]. Typical methods include particle swarm optimization, genetic algorithm, evolutionary algorithm, stochastic algorithm, game-based algorithm, and so on. In this paper, the network server (NS) is the center of star topology and is responsible for optimizing the data transmission rate and the transmission energy of the end node (ENs), intending to optimize the network scalability tags, as shown in Figure 1. The label management software processes and packs the information about the product and the prices configured into packets of data. The record packets are then dispatched to the gateways through wireless technology. Once the packets are transmitted to the gateways, they are then dispatched to the terminal display to update the price labels, based entirely on the records inputted into the label control software program. LoRa network allows the display to be updated whenever the product price is changed. This communication network is the major differentiation that undoubtedly makes ESL a possible solution. The ESL hardware configuration generally includes the circuit design of two modules, the communication station and the terminal display.

Most of the research performed thus far is focused on the outdoor application of LoRa rather than indoor. Previous strategies sought to optimize the overall performance of LoRa for IoT devices rather than BIoT (Building Internet of Things). This research is based on improving the performance of the communication module, which includes its throughput, and acknowledgment techniques for BIoT. LoRa uses the LoRaWAN protocol and chirp modulation technique for data transmission. LoRaWAN is designed for powerless and low data communication purposes. LoRaWAN has several drawbacks. Increasing the number of ENs for the same SF increases the probability of collision which decreases the total throughput [4]. The encoding method and transmission power need to change as the distance between GW and EN increases [5]. LoRaWAN cannot be used for large data payloads and is limited to 100 bytes. Furthermore, it cannot be used for continuous monitoring (except Class C devices). Thus, it is not an ideal candidate for real-time applications that require lower latency and enclosed jitter. Duty cycle (DC) constitutes only 1% of the total time during which the channel can be occupied [6]. This parameter arises from the ISM regulation as the key limiting factor for traffic served in the LoRaWAN network, and the data packet having a longer transmission time than the duty cycle is lost. LoRaWAN uses a pure ALOHA mechanism, which can cause inefficiency due to not eliminating fast saturation even with an increasing number of gateways [7]. This decreases the throughput, which then leads to data loss. Pure ALOHA allows the gateways to transmit the data at any time and, after transmitting, needs to wait for some time. After receiving acknowledgment from the receiver side, the transmission is considered successful and vice versa, while slotted ALOHA divides the time of the shared channel into discrete time slots, and any gateway is allowed to transmit the data using any time slot. For a low load, the delay in pure ALOHA’s channel is less than that in the slotted ALOHA’s channel. If the channel load is low, then the chances of collision in pure ALOHA will experience less delay for the gateway to transmit and retransmit. In slotted ALOHA, the system must wait for the next time slot. The slotted ALOHA is better than the pure ALOHA for a normal load as the probability of collision is lower in comparison [8]. The limitation of DC in slotted ALOHA and the lower throughput in pure ALOHA decrease the performance of LoRa-based ESL. To overcome these problems, some mechanisms have been used.

## 2. Related Work

Sungryul Kim optimized the pure ALOHA with the gradient projection method for optimal distribution of spreading factor (SFs) by using LoRaWAN with FHSS (frequency-hopping spread spectrum) to mitigate the collision problem by using a smaller SF, and the maximum number of ENs can achieve a maximum throughput of 62%, while ignoring the downlink DC [4]. Gyubong Park sought to improve the throughput as well the energy efficiency. This implementation was based on reinforcement learning only for 20 data samples, where the algorithm took a long time to learn. This method was suitable for very low data rates and very long transmission. Thus, the energy efficiency was improved but the throughput was decreased by up to 15% [5]. Dimitrios Zobras analyzed the same SF transmissions in different time slots and different SF transmissions in parallel by allocating SFs to nodes while maintaining the duty cycle [6]. According to Jetmir Haxhibeqiri, when the number of nodes increases up to 1000 per gateway, pure ALOHA will experience around 90% losses. However, LoRaWAN will reach up to 32%. Lowering the DC of the application layer below the allowed radio duty cycle of 1% would decrease the losses [7]. Tommaso Polonelli introduced a synchronized ALOHA protocol that uses clock synchronization across end nodes to define the slots for data packets for acknowledgment. The S-LoRaWAN was developed for low-power IoT devices with reduced costs. The packet collision was reduced to 26% for high traffic, and throughput up to 5.8× in real life by deploying 24 nodes operating for hours, theoretically doubled the throughput of the network [8]. Andri Rahmadhani showed the various aspects of collision in LoRaWAN for a single gateway [9]. In Ferran Adelantado’s study, due to latency, jitter, or reliability constraints, the pseudorandom channel hopping method was used in LoRaWAN to distribute transmissions of available channels, which was enough to reduce the collision probability. To minimize the collision, new pre-adoptive hopping methods have been introduced. Time division multiple access (TDMA) was used for the proposed channels to sequence both uplink and downlink with acknowledgment [10]. Ruki Harwahyu developed a multichannel slotted aloha for IoT based on a random access procedure by using an iterative contending user estimation model [11]. Haiyan Hu introduced an anti-collision algorithm (CD-ALOHA); the throughput of CD-ALOHA was better than that of the slotted ALOHA and CSMA algorithm [12]. Michele Zorzi performed the execution of LoRa in NS3 by using a pure ALOHA scheme and CSMA and CSMA-10 to lower the collision and power consumption in LoRaWAN [13]. Nevertheless, LoRa lacks some features needed to develop a real-time communication network. The uplink messages of LoRaWAN at the network server are not shown to the nodes by default. It is useful to have ACKs for nodes, and the packet loss can be detected for retransmission easily. Semtech innovated LoRa in 2015 but did not disclose all the features to the public.

## 3. Methodology

The methodology includes improving pure ALOHA to optimize the throughput using an acknowledgment synchronization mechanism and the allocation of ENs for SX1276.

### 3.1. Improved ALOHA

**Figure 2:**
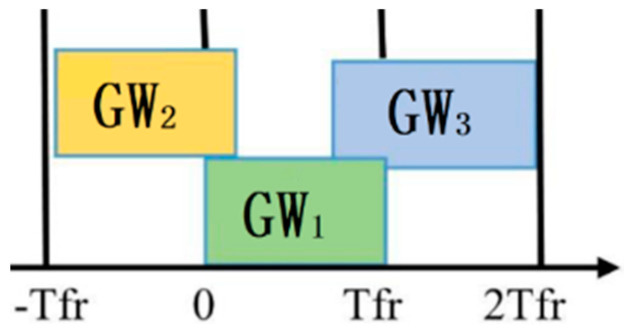
Pure ALOHA.

For pure ALOHA, if GW_1_ transmits a frame at the same instant (t = 0) or interval as other GWs, they will collide with each other. Figure 2 shows the length of time in which a collision can occur. Thus, 2Tfr is the vulnerable time in which every GW is allowed to transmit data whenever they are available. Without checking the status of the channel, every GW transmits data, which creates the possibility of data frame collision. ACK is received only for the delivered frame, not for collided or overlapped frames. The GWs wait for a random period of time for the damaged frame and try to retransmit the frame until it transmits successfully. To avoid a further collision, the waiting time of each GW must be random and different. The throughput of pure ALOHA is optimized when the frames are of the same length. The efficiency of this channel is the probability of successful transmission without any collision. The channel throughput of pure ALOHA is *η* = *Ge*^−2*G*^, where *G* is the number of GWs that wish to access the channel in one frame time, i.e., 1Tfr.

To derive the efficiency of the pure ALOHA channel, maximize *η* for *G*.

$$\begin{array}{l} d\eta /dG=0\Rightarrow d(Ge^{-2G})/dG=0\\ \Rightarrow -2Ge^{-2G}+e^{-2G}=0\\ \Rightarrow e^{-2G}(1-2G)=0\\ \Rightarrow e^{-2G}=0\;or\;1-2G=0\\ \Rightarrow G=\infty ,\;or\;G=1/2 \end{array}$$

where the finite value of *G* is used for examing the throughput of the network. Thus G means that half of the GWs need to transmit in a Tfr. For maximum efficiency, essentially one GW should transmit in 2Tfr. The maximum efficiency of pure ALOHA is 18.39%.

$$\begin{array}{l} \eta =Ge^{-G}\Rightarrow \eta =1/2e^{-2\times 1/2}\\ \eta =1/2e^{-1}\Rightarrow \eta =0.1839 \end{array}$$

For slotted ALOHA, each GW is allowed to start the transmission only within a discrete time slot. The time slot is t = 0, ±Tfr, ±2Tfr …, where Tfr represents one time slot for each frame. In contrast to the pure ALOHA, the slotted ALOHA does not allow data transmission whenever the GW has already data to send. In slotted ALOHA, the GW needs to wait till the next time slot begins and then permits each data frame to be transmitted in the new time slot.

**Figure 3:**
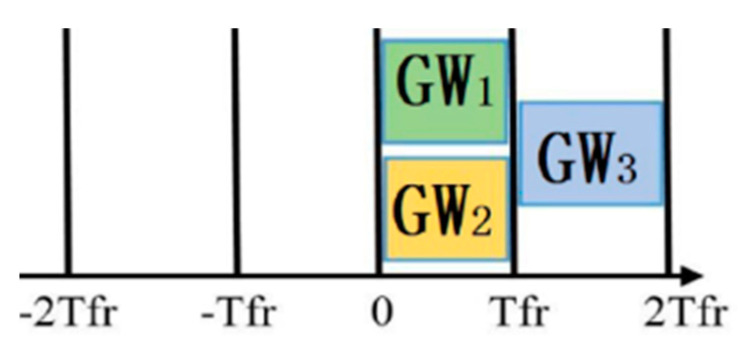
Slotted ALOHA.

For synchronization, the slotted ALOHA uses a special GW that emits a noise at the beginning of every time slot as a clock. In slotted ALOHA, 37% of the time slot is empty, which leads to a bandwidth loss, with 37% successes and 26% collision. Figure 3 shows that frames using the same time slot during transmission will collide. Thus, the vulnerable time is Tfr. In the case of slotted ALOHA, the efficiency is *η = Ge^−G^.*

$$\begin{array}{l} d\eta /dG=0\\ \Rightarrow d(Ge^{-G})/dG=0\\ \Rightarrow -Ge^{-G}+e^{-G}=0\Rightarrow e^{-G}(1-G)=0\\ \Rightarrow e^{-G}=0,\;or\;1-G=0\\ \Rightarrow G=\infty ,\;or\;G=1 \end{array}$$

where the finite value of *G* represents the number of GWs that want to access the channel within one Tfr. The infinite value of *G* is ignored. Therefore, the total number of requests is made per time slot, which is also called the channel load. We can achieve the maximum efficiency when a single GW accesses the channel per time slot.

$$\eta =1\times e^{-1}\Rightarrow \eta =0.3678$$

It is justified that the efficiency of slotted ALOHA is twice that of pure ALOHA. Thus, the throughput is also double [6,7,8]. However, LoRa defines the same system as pure ALOHA by using a blind transmission technique to transmit the frame without channel sensing. ALOHA for a large LoRa network uses the Poison process with the average rate of *G* attempts per slot. Keeping an equal packet length, the ENs with the same SF will collide; then, *G* is the average attempt per time slot.

(1) $$G=GW_{n}\times \rho_{t}\times \lambda_{t}\times ToA_{t}\times EN_{n}$$

Let $EN_{n}$ represent the number of *ENs* using $SF_{t}$ with packet generation rate $\lambda_{t}$, while $\rho_{t}$ is the probability of EN using $SF_{t}$ and $GW_{n}$ is the number of gateways that attempt to transmit. ToA is the time-on-air from GW to EN. The range of CF (carrier frequency) is 137 MHz to 1020 MHz using SF7 to SF12. The power directly affects the quantity of energy used to transmit a chirp, while the timing, which depends on the D between the EN and GW, affects the throughput of the LoRa network. To solve this problem, the LoRa network is designed based on orthogonal combination principles. In Table 1, “X” is not the orthogonal combination of SF and bandwidth (BW). SF in LoRa is the ratio of the RS (symbol rate) and the RC (chip rate), ${\mathrm{SF}=\log}_{2}\left(\mathrm{RS}/\mathrm{RC}\right)$. BW would also determine the chirp duration. Therefore, different SFs will give different slopes. Not all combinations are orthogonal because some of them define the same chirp rate, which results in the same slope [8,14,15,16]. Thus, SF7/BW125kHz has the same chirp rate as SF7/BW250kHz (122070312.5), SF8/BW125kHz has the same chirp rate as SF10/BW250kHz, SF9/BW125kHz has the same chirp rate as SF11/BW250kHz, SF10/BW125kHz has the same chirp rate as SF12/BW250kHz, and so on.

Due to the restriction of duty cycle and dependence on ToA, the frame generation rate is $\lambda_{t}=DC/ToA_{t}.$

(2) $$ToA_{t}=T_{preamble}+T_{payload}+CRC+T_{Symbol}$$

where $T_{preamble}$ is the preamble duration and $T_{payload}$ is the payload duration, which also includes a header and cyclic redundancy check (CRC) fields, which are optional. The main part is the preamble, which consists of a sequence of two down-chirps, constant up-chirps, and a quarter of up-chirps. The preamble is used by the receiver to start synchronizing with the transmitter. SF and BW have a direct impact on the ToA of the LoRa packet because these parameters are typically defined as the symbol rate. Thus, higher SF increases ToA, and higher BW decreases ToA at the cost of the receiver sensibility.

Thus,

(3) $$\eta_{t}=EN_{n}\times GW_{n}\times \rho_{t}\times \lambda_{t}\times ToA_{t}\times e^{-2\times EN_{n}\times GW_{n}\times \rho_{t}\times \lambda_{t}\times ToA_{t}}$$

where $e^{-2G}$ is the probability of successful transmission *P* (0 ≤ *P* ≤ 1) for a frame. For the LoRa network, it is

(4) $$P_{t}=e^{-2\times EN_{n}\times GW_{n}\times \rho_{t}\times \lambda_{t}\times ToA_{t}},t\in \;SF$$

Furthermore, the outage probability is $P_{{out}_{t}}=1-P_{t},\;t\in SF$.

Algorithm 1 displays the working principles where, initially, the locality of the EN is obtained by using RSSI estimation and SF allocation. The ρ, which depends on the combinations of GWs and ENs, helps to minimize the saturation while using the Poison process for packet generation. The network server controls the BW, which depends on the DC and saturation during the transmission. The transmissions taking a longer time than the normal operation or with higher saturation are subjected to NS.
**Algorithm 1.** ALOHAInput: ENs, GWs, D, DC Initialize: t, λ, BW, RSSI Ensure: η **for** *t* = 7,8,…,12 **do** Calculate ENs’ RSSI for each cluster via Table 5 Estimate ENs’ ρ using each combination of GWs via Table 6 **if** (DC_Limit_ > DC) and (ToA_MLA_ > ToA_t_) **then** GW transmits data to EN **else if** (DC == DC_Limit_) & (ToA_MLA_ > DC) **then** Higher the BW **else if** (Sat._MLA_ > Sat._Limit_) & (ToA_MLA_ < DC) **then** Lower the BW **else** alert unsuccessful transmission **end if** **end for** ACK==ACK_Limit_ GW synchronizes EN

### 3.2. Improved Acknowledgment

**Figure 4:**
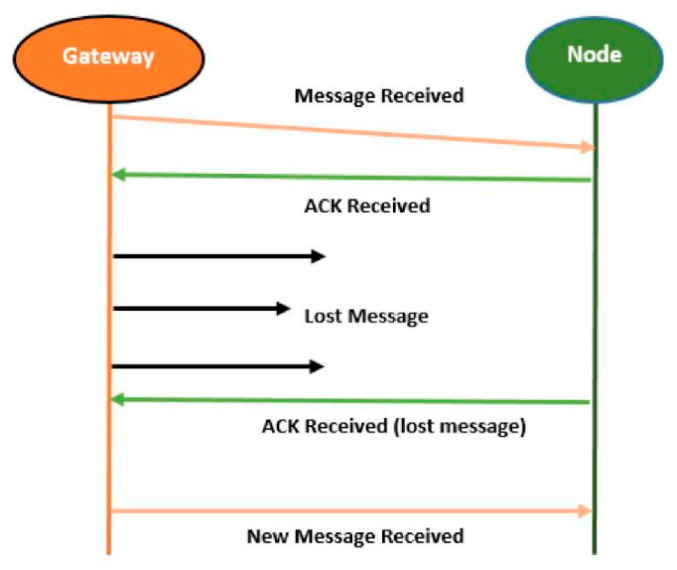
Failure of message integrity code.

After several unsuccessful transmissions by the GW, the EN will wait for retransmission. However, due to the ACK drop at the GW, the GW will send a new message to the EN while the EN will contact the ACK for the lost message. The chaos between GW and EN leads to improper implementation of ACK’s message integrity code (MIC), as shown in Figure 4. Due to the half-duplex nature of the GW, it is not capable of listening to the channel during the uplink and downlink at the same time. The frame transmission interval and transmission response cannot be handled in this specific DC (pure ALOHA). Selecting a random delay between 1 and 3 s for ACK is not precise, and if the duration of a frame and an ACK are more than a second, this will result in a high probability of repeated collision with a frame or ACK. An increase in the random delay, which is a fixed interval (slotted ALOHA), will limit the network scalability. Any two transmissions overlapping in time at the EN with the same CF, SF, and BW will collide and be lost.

**Figure 5:**
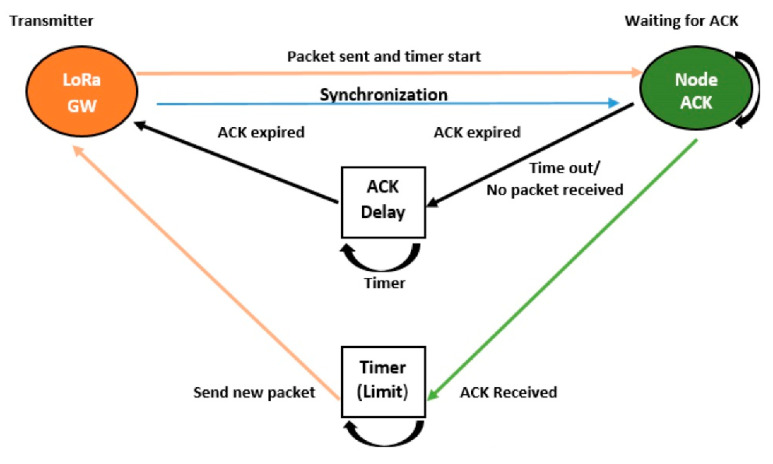
Synchronization acknowledgment.

Thus, to handle this problem, the EN is synchronized to the GW’s clock by subtracting the ToA of the payload from the ACK’s locality delay and vice versa. If the packet is lost, the GW will not retransmit it but will send six symbols of the preamble to resynchronize the EN, as shown in Figure 5 [16,17].

Algorithm 2 represents the ACK technique, which is based on synchronization rather than retransmission. The ToA and ACK’s time depend on the locality of the EN. Thus, ENs at higher SFs have higher synchronization times as compared to ENs at lower SFs. The same strategy is applied to both uplink and downlink.
**Algorithm 2.** AcknowledgmentInput: DC, D Initialize: ENs/SF to GW via Table 2 ACK_Limit_ = ToA + ACK’s time + Synchro. via Figure 5 Ensure: ACK_CNT_ = 0 for all ENs in the range of GW **for** UL transmission **do** ACK_CNT_ = ToA + ACK’s time ACK not received then ACK_CNT_== ACK_Limit_ NS sends six symbols of preamble for Synchronization via Table 3 **end for** **for** ACK_CNT_ ≥ ACK_Limit_ + Synchro. **do** RSSI Estimation to find the locality of EN via Table 4 **end for** **for** DL transmission received **do** ACK_CNT_ = 0 **end for**

## 4. Implementations and Results

This section includes configurations and conditions, as well as implementations and results.

### 4.1. Configurations and Conditions for ALOHA

As the number of obstacles and the distance to the gateway increase, the RSSI values decrease. This behavior is observed for all SF values. It is expected that, theoretically, the minimum RSSI for received packets decreases with an increment in the SF value. For strong signals under better reception conditions, the value of SNR rarely exceeds 8dB. Thus, the SNR (signal to noise ratio) is not a good indicator of signal quality for stronger signals. According to practical experiments, when SNR approaches the limit specified for the spreading factor (SF8), then the packet reception will start to fail. We need to consider the downlink capacity of the network before starting communication. A LoRa gateway can take responsibility for communicating with hundreds of ENs. If the gateway is constantly transmitting data, on the demand of every EN, it will raise the noise floor and consume more time that could be used for uplinks. Thus, we used a different number of gateways and measured the RSSI to improve the performance of the network based on K-mapping, using NS3 (Network Simulator) for the simulation work and the LoRa calculator for confirming the results [1,18,19]. Basic parameter selection for this research work is given in Table 2.

### 4.2. Implementations and Results

The first simulation includes the implementation of a GW without using MLA for 100 ENs/SF and 200 ENs/SF, respectively, to analyze the performance of LoRa for ESL. The CRC (4/5) does not affect the ToA at SF8, SF10, and SF11. Table 3 represents the corresponding bit rate, ToA, T_preamble_, T_symbol_, and T_paylaod_ for a 16-byte payload with six symbols of the preamble for a single gateway at the DC of 2000 ms. Thus, at SF12, the GW and ENs have enough time to properly communicate.

**Figure 6:**
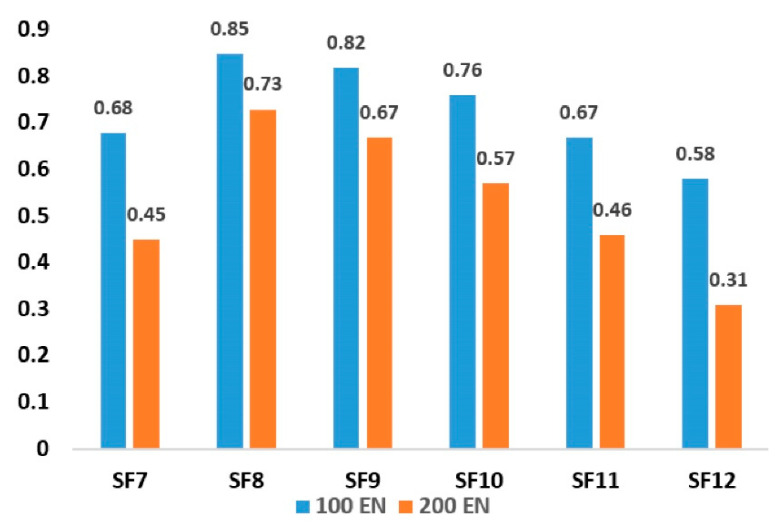
Successful transmission for a gateway.

For simplicity, we did not use both BW125 and BW250, in order to ensure that the different values of SF did not interfere in the measurement of the limit of successful transmission of LoRaWAN. The probability of successful transmission depends on the values of SF and ENs using 125 kHz, which are given in Figure 6. An increasing number of ENs diminishes the performance of the network at higher SF values. Initially, from SF7 to SF8, the possibility of successful transmission increases and then decreases to the minimum possible successful transmissions at SF12. One of the reasons for this is that the devices need a great deal of time to move from the lower to the higher value of SF to regain connectivity, and this process requires the devices to lose a sufficient number of sent packets before moving to a higher SF. This behavior is obvious for static devices, as is most likely the case in a large variety of IoT (Internet of Things) deployments [1,8,20].

To find a solution to this problem, a specific SF value is allotted to each EN depending on its distance from the GW based on K-means clustering.

(5) $$C=\underset{GW_{n}\in C}{\arg\;\min}\frac{1}{\left|EN_{n}\right|}\sum_{EN_{t}\in EN_{n}}dist^{2}\left(GW_{n},EN_{t}\right)$$

where *C* is the 2-D vector of border points around the Cartesian coordinates of the centroids, which are determined by the algorithm. The function $dist.\left(x_{1},\;x_{2}\right)$ is the Euclidean distance between $x_{1}$ and $x_{2}$. The ENs in the centroid boundary forming the set $R=\left[R_{x},R_{y}\right]$ are separated and act as vectors storing the coordinates of the inner ENs for each of the Cartesian dimensions. The maximum absolute value of each dimension of *R* is calculated to set the radius $R_{t}=max\left|R_{x}\right|+max\left|R_{y}\right|/2$ for defining the limit of the ring for SF7 to SF12. The procedure is repeated to determine the boundaries of the remaining SF rings ($R_{5}\;to\;R_{1}$).

When changing the SF at each step from the lower value to the higher, double ToA is needed to transmit the same data packet. ToA is changed as the location of the ENs is arranged based on the K-means MLA. RSSI estimation is introduced to determine the exact location and energy demand of the ENs. RSSI is the total signal power received in decibel-milliwatts (dBm). For LoRa, it is often represented in negative dBm, which means that a value closer to 0 indicates a better signal.

(6) $$RSSI_{\left(dB\right)}=P_{{TX}^{\left(dBm\right)}}+G_{{TX}^{\left(dBi\right)}}-L_{{PL}^{\left(dB\right)}}$$

This part of the simulation includes the RSSI estimation of a GW for 200 ENs/SF to determine the exact position of the ENs, which helps to check the performance of the LoRa network for single and multiple GWs. The value of RSSI indicates the quality of communication between the GW and EN. An RSSI of −108 dBm is considered good while −124.6885 dBm is considered poor for a GW based on our simulation work, as shown in Table 4. The ToA is higher as compared to the results obtained without MLA.

As the RSSI for a GW is useful in determining the real position of the ENs, the RSSI estimation for multi-gateways at a distance ranging from 0.7 km to 2.1 km will also help to determine the exact position and energy harvesting of the ENs from the gateways at each SF, which is shown in Table 5. An increasing number of GWs improves the RSSI but does not guarantee the maximum possibility of successful transmission. Receiving signals carrying the same data packet from different GWs causes congestion at the EN.

Due to orthogonality principles, we cannot use the non-orthogonal combination of SF and BW. Thus, this part of the simulation is divided into two parts including the individual implementation of SF/BW125 and SF/BW250. A BWS indoor long-range propagation model BIoT for SX1276 using K-mapping for clustering is developed. Besides the orthogonal combinations of SF and BW, DC is also an important factor in running a smooth network. Each EN has a specific communication characteristic, defined by the transmission parameters SF, D, and GW. For an experiment, GW’s transmission behavior is described by the average packet transmission rate λ = 1/DC, and a packet payload of 16 bytes. The preamble length is 6 symbols, and the ToA for a packet is given by payload, SF, BW, and CR. The probability of ENs using different GWs decreases with the increasing number of GWs, as shown in Table 6.

**Figure 7:**
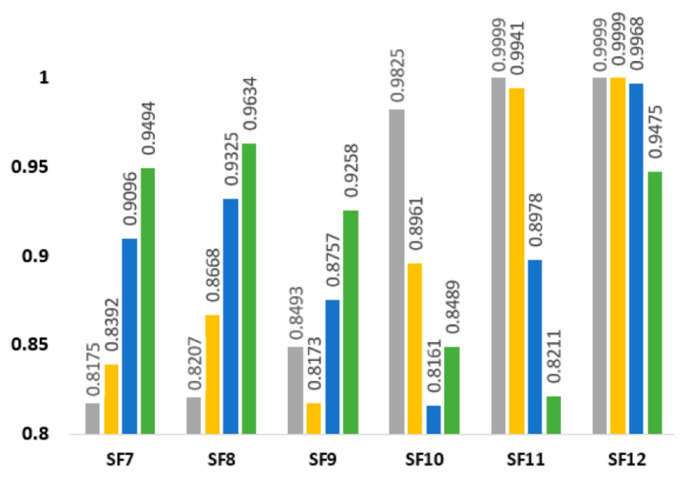
Multi-gateways using K-means MLA (125 kHz).

After determining the key parameters for successful transmission, the behavior of 200 ENs/SF during simulation is therefore described by the ρ, SF, BW, λ, D, and GWs. Each EN can receive data for a given CF with multiple signals with different combinations of SF and BW. The overall throughput suggests that for mid- and long-range ENs, increasing the gateways improves the performance of the LoRa network anonymously, as shown in Figure 7.

The DC is 2000 ms, which is higher than normal operation (1000 ms), and the ENs at the mid and high range have enough time to receive the packets. The sensitivity of this parameter selection is high for both GW and EN. The drawback of this implementation is the slow transfer of data (16-byte payload) [21,22,23,24]. For ESL, fast and frequent updates of ENs are required. However, as many ENs in the LoRa network can converge to use the same LoRa parameters, the probability of collision and overlapping may increase, leading to lower link quality, thus impacting the convergence time. We changed the DC to 1000 ms and BW to 250 kHz in order to enlarge the frequency range (CR4/5) and recheck the performance of the system, as shown in Table 7.

**Figure 8:**
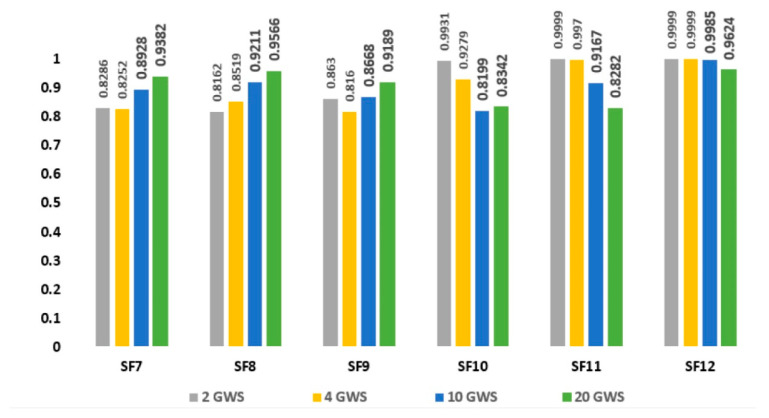
Multi-gateways using K-means MLA (250 kHz).

The traditional method doubles the bit rate and halves the ToA, which is the main cause of the saturation. The K-mapping of the ENs estimates the locality of the ENs by measuring their RSSI. Moreover, a minor increase in the ToA helps the ENs to receive the packet within the time frame, which has a direct impact on improving the sensitivity of ENs. The bit rate and BW are the same as in the traditional method. Figure 8 illustrates that each combination of GWs has different performance for the low-, mid-, and long-range ENs. The higher combinations of GWs perform better for the ENs at SF7, SF8, and SF9, while lower combinations of GWs perform better for ENs at SF10, SF11, and SF12 [24,25,26].

The increasing BW reduced the possibility of successful transmission because the faster data transfer causes saturation at the receiver side. Thus, the LoRa network has more congestion at a higher BW as compared to a lower BW for traditional algorithms. To minimize the saturation to a certain degree, we used K-means MLA. Table 8 shows the percentage of overall saturation for both methods.

Table 9 displays the battery lifespan in days for the case in which the EN has a 3.3-volt battery with 1000 mAh capacity and interrogates twice per day. The DC and the number of interrogations between EN and GW have a greater impact on the battery life of the EN. Decreasing the DC shortens the life span and vice versa. The increasing number of frequent updates also increases energy harvesting.

## 5. Conclusions

This research aims to perfect the pure ALOHA for LoRa-based ESL. During the simulation, the increasing number of GWs using MLA leads to different behavior as compared to a single GW. Without MLA, the performance of the network degrades for a GW with increasing SF and ENs, while for multi-GWs, the performance of the network improves by using K-mapping. Decreasing the BW boosts the network to the highest successful transmission rate but leads to higher ToA and $T_{preamble}$, which slows down the network. Moreover, the packets taking a longer time than the DC are rejected. The higher BW doubles the data transfer and halves the $T_{preamble}$, which improves the synchronization but causes saturation at the ENs without MLA. The DC has a significant impact; at a BW of 125 kHz, the packet has ample time to be received by the ENs, which enhances the time to deliver a payload of 16 bytes, which is unacceptable in a large ESL deployment. BW125 kHz has higher energy consumption as compared to BW250 kHz at the same DC; thus, the ENs would have a short battery lifespan for a lower BW. Using the BW250 kHz at lower SFs improves the battery lifespan of the ENs. There is a trade-off between the number of gateways as well as network performance. The performance of the network is not optimistic for lower SFs as compared to higher SFs. Minimizing saturation at a lower SF for higher BW and collision at a higher SF for lower BW is the most challenging task because using lower and higher BW at the same time causes degradation due to the orthogonality principle. Furthermore, if better battery lifespan and performance are the key aspects, then the number of gateways should be increased, which directly increases the installation cost as well as the network complexity. Future work should include a saturation graph and the demonstration of hundreds of ENs using a few GWs with practical results.

## Figures and Tables

**Table 1:** Orthogonal combinations of SF and BW.

**SF**		7	8	9	10	11	12	7	8	9	10	11	12
	**BW**	125	125	125	125	125	125	250	250	250	250	250	250
7	125	X								X			
8	125		X								X		
9	125			X								X	
10	125				X								X
11	125					X							
12	125						X						
7	250							X					
8	250								X				
9	250	X								X			
10	250		X								X		
11	250			X								X	
12	250				X								X

**Table 2:** Parameter selection.

Parameter	Value
CF	868 MHz
BW	125 kHz, 250 kHz
SF	7–12
Transmission power (PTX)	14 dBm
Payload	16 bytes
ENs/SF	100, 200
GWs	1,2,4,10,20
Medium loss (LPL)	127.84 dB
Gain (GTX)	2.15 dBi
DC	2000 ms, 1090 ms, 1000 ms

**Table 3:** Performance of a gateway.

SF	Bit Rate (bps)	ToA_Payload_ + CRC (ms)	ToA_Payload_ (ms)	T_Preamble_ (ms)	T_Symbol_ (ms)	ToA_t_ (ms)
7	5468.75	44.29	39.17	10.50	1.02	55.81
8	3125	78.34	78.34	20.89	2.05	101.28
9	1757.81	156.67	136.19	41.98	4.10	202.75
10	976.56	272.38	272.38	83.97	8.19	364.54
11	537.11	544.77	544.77	167.94	16.38	729.09
12	292.97	1089.54	925.7	335.87	335.8	1761.21

**Table 4:** Single gateway based on K-means MLA.

SF (125 KHz)	D (km)	ToA_MKA_ (ms)	Receiver RSSI (dBm)	Received RSSI (dBm)
7	0.7	59.65	−109	−108.6651
8	0.9	98.82	−112	−112.0519
9	1.1	177.15	−115	−115.0773
10	1.5	354.3	−118	−118.4322
11	1.6	626.69	−120.5	−120.8336
12	2.1	1253.38	−123	−124.6885

**Table 5:** RSSI estimation of multi-gateways.

SF	D (km)	2 GWs RSSI (dBm)	4 GWs RSSI (dBm)	10 GWs RSSI (dBm)	20 GWs RSSI (dBm)
7	0.7	−112.2239	−111.242	−107.096	−107.551
8	0.9	−115.8398	−110.644	−111.835	−112.139
9	1.1	−115.5934	−112.924	−112.996	−114.134
10	1.5	−116.3864	−118.250	−116.812	−116.040
11	1.6	−120.7323	−119.240	−119.912	−120.090
12	2.1	−121.8479	−122.614	−126.787	−104.131

**Table 6:** Probability of ENs using GWs.

SF	D (km)	1 GWs	2 GWs	4 GWs	10 GWs	20 GWs
7	0.7	0.18	0.095	0.048	0.019	0.009
8	0.9	0.08	0.040	0.020	0.008	0.004
9	1.1	0.11	0.055	0.275	0.011	0.0055
10	1.5	0.15	0.075	0.375	0.015	0.0075
11	1.6	0.21	0.100	0.050	0.020	0.0100
12	2.1	0.30	0.140	0.070	0.028	0.014

**Table 7:** Multi-gateways based on K-means MLA.

SF (125 kHz)	Bit Rate (bps)	ToA_Payload_ + CRC	ToA_MLA(Payload)_	T_Preamble_ (ms)	T_Symbol_ (ms)	ToA_MLA_ (ms)
7	10,937.5	22.14	37.5	5.25	0.51	43.26
8	6250	39.17	59.65	10.50	1.02	71.17
9	3515.63	78.34	98.82	20.99	2.05	121.86
10	1953.13	136.19	218.11	41.98	4.10	264.19
11	1074.22	272.38	354.3	83.97	8.19	446.46
12	585.94	544.77	708.61	167.94	16.38	892.93

**Table 8:** Saturation comparison.

SF	Saturation 125 kHz	MLA Saturation 125 kHz	MLA Saturation 250 kHz
7	33.06	12.11	12.88
8	25.49	10.42	11.36
9	36.33	13.30	13.38
10	46.95	11.41	10.62
11	59.13	7.72	6.50
12	57.83	1.40	0.98

**Table 9:** Energy harvesting comparison.

SF	MLA(DC = 2 s) 125 kHz	MLA (DC = 1 s) 250 kHz	MLA(DC = 1.09 s) 125 kHz
7	4731.5	3618.5	2384.7
8	2570.9	1916.1	1292.2
9	1325.8	965.3	665.4
10	664.6	474.0	333.3
11	328.5	230.0	164.7
12	161.21	111.01	88.01

## Data Availability

Not applicable.
